# Efficacy of hemoadsorption in the severe course of COVID-19

**DOI:** 10.3389/fmed.2025.1491137

**Published:** 2025-03-06

**Authors:** Aleksei Yurievitch Yakovlev, Yuri Vladimirovitch Ilyin, Feodor Feodorovitch Bershadsky, Dmitry Dmitrievitch Selivanov, Aleksei Aleksandrovitch Pevnev, Artur Igorevitch Trikole, Aleksander Yurievitch Popov, Vladimir Mitrofanovich Pisarev

**Affiliations:** ^1^Semashko N.A. Nizhegorodsky Regional Clinical Hospital, Nizhny Novgorod, Russia; ^2^Nesmeyanov А.N Institute of Organoelement Compounds of Russian Academy of Sciences (INEOS RAS), Moscow, Russia; ^3^Federal Research and Clinical Center of Intensive Care Medicine and Rehabilitology, Moscow, Russia

**Keywords:** extracorporeal therapy, hemoadsorption, interleukin-6, immunomodulation, multiple organ deficiency, severe COVID-19, viral sepsis

## Abstract

**Introduction:**

Insufficiencies of the majority of targeted therapies for the most severe, life-threatening forms of COVID-19 warrant alternative, adjuvant treatment options for enhanced life maintenance that include extracorporeal blood purification and homeostasis support. The goal of the current study is to evaluate the clinical efficacy of hemoadsorption with mesoporous hypercrosslinked polystyrene beads (Efferon CT single-use cartridge) in an expanded cohort of patients with severe and critical COVID-19 resistant to antibody therapies and requiring post-therapy invasive mechanical lung ventilation (MLV) versus parameter-matched control group with no hemoadsorption.

**Materials and methods:**

A single-center cohort study (NCT06402279) enrolled patients from October 2020 to February 2022: the Efferon CT group (non-responders to anti-cytokine antibody therapy requiring IMV, hemadsorption, and standard treatment, *n* = 65) and retrospectively acquired propensity-matched control group (no hemadsorption, standard treatment only, *n* = 65).

**Results:**

This observational study revealed the capability of Efferon CT hemoadsorption to safely, rapidly, and significantly reduce the need for norepinephrine, increase the oxygenation index, prevent the sepsis-associated AKI, decrease the development of multiorgan failure, and restore the immune system balance by decreasing pro-inflammatory IL-6, ferritin levels, and neutrophil-to-lymphocyte ratio.

**Conclusion:**

The clinical efficiency of hemoadsorption using Efferon CT was confirmed by the resolution of acute respiratory failure in 54% of patients, significantly increasing the number of days without mechanical ventilation and increasing early the index of oxygenation. Most importantly, the hemoadsorption with Efferon CT was safe and resulted in a significant decrease in the mortality of severe COVID-19 patients.

**Clinical trial registration:**

www.clinicaltrials.gov, Identifier NCT06402279.

## 1 Introduction

During the COVID-19 pandemic, most patients with mild and moderate forms of COVID-19 with oxygen saturation > 94% did not require hospitalization. A more severe course of COVID-19 is associated with excessive inflammatory responses causing endothelial cell damage, disbalance of the blood coagulation and anticoagulation systems, and disruption of the respiratory and urinary systems, commonly requiring hospitalization. The more severe disease course is characterized by the level of SpO_2_ below 94% and the ratio of arterial oxygen pressure to the fraction of inspired oxygen (PaO_2_/FIO_2_) < 300 mmHg with a respiratory rate > 30 breaths/min, or the presence of infiltrates in the lungs >50%. Patients with more severe courses develop critical illness associated with severe respiratory failure, increased SOFA values, and septic shock development (1). Further progression of COVID-19 is complicated by the acquisition of bacterial hospital-acquired infection (HAI) with the increased risk for bacterial sepsis and septic shock (2, 3) that lead to a high (up to 60%) mortality in ICU patients (4–6).

The lack or insufficient activity of most targeted therapies for life-threatening forms of COVID-19 warrants a search for alternative, primarily pathogenetic approaches to treatment that include extracorporeal blood purification and homeostasis support (hemodialysis, plasmapheresis, and hemofiltration) for more comprehensive life maintenance in the body (7–9).

Insufficient clinical effectiveness of filtration/adsorption methods for removing toxic and pathogenetically significant molecules from the body (10) of severe COVID-19 patients has led to the development and application of various hemoadsorption devices to adsorb low and medium molecular weight molecules, which exhibited damage-associated molecular patterns (DAMPs) and pathogen-associated molecular patterns (PAMPs) (11–14). Timely extracorporeal removal of PAMPs and DAMPs, including bacterial endotoxins, cytokines, and other medium molecular weight molecules using sorption technologies, has a high potential to counteract the escalation of decompensation of functions of vital organs occurring in severe cases of bacterial and viral sepsis, including COVID-19 (15–18). Accumulated experience in using hemoperfusion devices for blood purification in sepsis has confirmed the need to expand their technological diversity and improve the clinical significance of hemadsorption for severe COVID-19 cases (19–22).

However, the results of clinical studies on the effectiveness of hemoadsorption turned out to be contradictory, which is associated with the timeliness and characteristics of hemoadsorption protocols, differences in the timing of the start of treatment, heterogeneity of patients in terms of duration and stage of the disease, age, comorbid background, and severity of multiple organ failure (MOF) (23, 24).

A recent meta-analysis of 16 clinical trials utilizing hemoadsorption, comprising 86 patients with severe COVID-19 treated with CytoSorb®, oXiris®, Biosky filter, SeaStar® CLR filter, HA280, and HA330 Jafron© cartridges, further emphasized the potential of hemoadsorption with diverse adsorbents for the efficient management of patients with severe COVID-19 hyperinflammatory manifestations (25). Application of early hemoadsorption was associated with a reduction in mortality, shorter duration of MLV, ECMO, and ICU stay, and high levels of safety performance (26, 27). Similar trends for clinical effects and outcomes were observed when novel mesoporous polymeric matrix Efferon CT cartridges were employed for hemoadsorption in patients with rhabdomyolysis (28) and a small group of patients diagnosed with COVID-19 (29).

The clinical effectiveness of hemoadsorption using the hemoadsorption device Efferon CT in patients with more severe forms of COVID-19, who are resistant to antibody therapies and require post-therapy invasive mechanical lung ventilation (MLV), has not been studied. However, the use of a similar polymeric adsorption matrix in bacterial septic shock in adult and pediatric sepsis patients in recent studies confirmed its enhanced potential in the removal of interleukin-6 (IL-6), procalcitonin, and C-reactive protein (CRP), as well as a decrease in the severity of multiple organ failure (MOF) and time to weaning from mechanical ventilation (30–33).

In this study, we evaluated the clinical efficacy of hemoadsorption using Efferon CT in an expanded cohort of patients with severe COVID-19 course resistant to anti-IL-6 therapies and required post-therapy MLV versus a matched control group with no hemoadsorption.

## 2 Materials and methods

### 2.1 Study design

A single-center cohort study was conducted at the N.A. Semashko Regional Clinical Hospital (Nizhny Novgorod, Russia) from October 2020 to February 2022 in accordance with the WMA Declaration of Helsinki—Ethical Principles for Medical Research Involving Human Subjects (34). The study was approved by the local independent Ethics Committee, Protocol No. 9 (dated 25 October 2020), and informed consent from patients had been received prior to inclusion in the study. The study protocol is available at https://clinicaltrials.gov/study/NCT06402279. For patients in the prospective group who could not provide informed consent themselves or if their legal representative was unavailable, the decision to include the patient in the study was made at the bedside by two physicians and the deputy Chief physician. This process was conducted in accordance with the study regulations approved by the Chief physician on 25 October 2020. From patients who regained consciousness, informed consent was obtained for the continuation of their participation in the study and the further use of their research data. In accordance with the observational study design, the retrospective control group was included in the study without obtaining informed consent, as permitted by Paragraph 32 of the Declaration of Helsinki (34), following approval of the local ethics committee.

### 2.2 Patients

**Figure 1:**
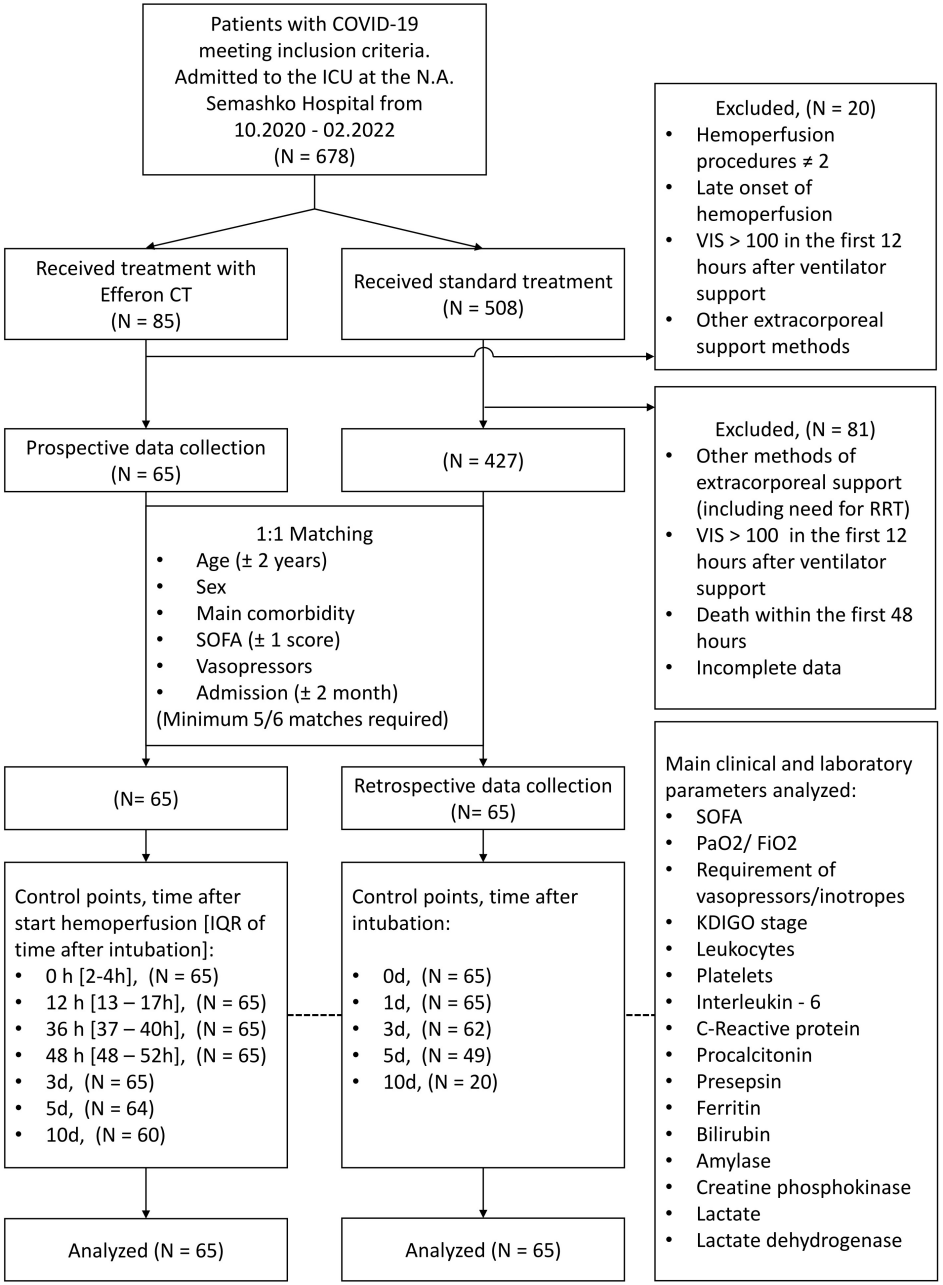
Trial flowchart.

The study included 130 patients with severe and critical course of COVID-19. The selection of patients for the study was performed based on a failure of anti-cytokine/cytokine receptor antibody treatment, the ineffectiveness of high-flow oxygen therapy and non-invasive ventilation, and the requirement for the invasive MLV (Figure 1). Each participant was included in the study during the first 6 h after transferring to invasive MLV. Inclusion criteria are as follows: severe or critical form of COVID-19 or severe critical condition as a result of this disease, age over 18 years and under 75 years old, non-pregnant patients or those who are 3 months or more postpartum, absence of acute bleeding, cancer (including anamnesis), thrombocytopenia less than 100 × 10^9^/L, acute cerebrovascular accident or acute myocardial infarction during the last 6 months and before the diagnosis of COVID-19, presence of a comorbidity index of no more than 5 points on the Charlson scale, and absence of previously performed extracorporeal detoxification procedures.

All patients received basic drug therapy for COVID-19 as per the methodological recommendations of the Ministry of Health of the Russian Federation: All patients received anti-cytokine drugs, glucocorticoid therapy (dexamethasone 16–24 mg/day), oxygen therapy, and heparin therapy. Inclusion in the Efferon CT group and transfer to the intensive care unit (ICU) for extracorporeal hemoadsorption was performed 2–5 days after initiation of treatment that demonstrated insufficiency of non-invasive MLV and combined drug treatment (Table 1).

**Table 1:** Demographic, clinical, and laboratory characteristics of patients in the compared groups at the time of inclusion in the study.

Parameter	Efferon CT group, *n* = 65	Control group, *n* = 65	*p*-value
Age, year	61 (53;67)	63 (55;67)	0.784
Gender, male/female	29/36	32/33	1
Comorbidity			
Hypertensive heart disease	43/65 (66%)	44/65 (67%)	1
Obesity	14/65 (21%)	13/65 (20%)	1
Coronary heart disease	16/65 (24%)	13/65 (20%)	0.674
Without comorbidities	3/65 (5%)	3/65 (5%)	1
One or more other comorbidities	12/65 (18%)	8/65 (12%)	0.345
Duration of illness			
From first syndromes to hospitalization, day	11 (9; 12)	10 (9; 12)	0.742
Pre-ICU hospitalization, day	4 (2, 5)	3 (3, 4)	0.435
ICU-pre-MLV, day	2 (2; 3)	2 (2; 3)	0.849
Clinical characteristics			
Volume of lung lesions according to CT scan during hospitalization, %	65 (55; 75)	65 (60; 70)	0.463
SOFA, score	4.0 (3;5)	4.0 (4;5)	0.770
Body mass index	26 (23; 35)	27 (24; 34)	0.956
PaO_2_/FIO_2_	126 (116; 134)	126 (113; 136)	0.635
Leukocytes, ×10^9^/L	11.4 (9.3; 14.5)	12.04 (9.6; 15.3)	0.570
Neutrophil-to-lymphocyte ratio	16.8 (13.3; 21.8)	15.6 (13.4; 19.1)	0.414
Vasopressor support, *n* (%)	14/65 (21%)	15/65 (23%)	1
AKI KDIGO I	5/65 (7.7%)	4/65 (6.2%)	0.746
Renal replacement therapy, *n* (%)	0/65 (0%)	0/65 (0%)	1
Anti-cytokine therapy, *n* (%)	65 (100%)	65 (100%)	1

Importantly, the relatively brief duration of both pre-MLV periods of hospitalization (pre-ICU and ICU-pre-MLV, Table 1) mirrors the rapid progression of the disease in a group of severe COVID-19 patients further requiring MLV. No differences were found between the control and Efferon CT patient groups (Table 1).

Antibacterial therapy in patients began from the day of transfer to invasive MLV. It included third-generation cephalosporins with subsequent adjustments based on the results of bacterial cultures and clinical and laboratory dynamics of indicators of the activity of bacterial infection.

Demographic, anthropometric, and clinical parameters data were evaluated when patients were transferred to invasive MLV and included in the study (Table 1). The severity of organ dysfunction was monitored using the Sequential Organ Failure Assessment (SOFA) scale; hemodynamics and gas exchange parameters were evaluated by determining mean arterial pressure (MAP), the use of vasopressor drugs, and the oxygenation index (PaO_2_/FIO_2_ Ratio, Horowitz index, the ratio of oxygen partial pressure in arterial blood in mmHg to the inspiratory oxygen fraction, respectively). At a baseline and every 24 h after inclusion in the study, the concentrations of leukocytes, lymphocytes, band neutrophils, platelets in the blood, and lactate levels, presepsin, procalcitonin (PCT), and interleukin-6 (IL-6) in plasma were determined. Microbiological monitoring was performed starting from the time of inclusion in the study; the duration of invasive mechanical ventilation, the need for renal replacement therapy (RRT), and hospital mortality were monitored up to day 60 of the study.

Blood, urea, and sputum were collected in sterile containers and properly labeled by experienced and trained nurses of the ICU and hospital according to intensive practices they had received during educational courses that employed contemporary internationally recognized practices of clinical sample collection and processing. Following the transfer of a patient to invasive MLV, sputum specimens for bacterial analyses were collected from bronchial segments by bronchoalveolar washing during a bronchoscopy. When the patient was given MLV in the prone position, no sputum was collected. Blood specimens were collected from a central venous catheter. To prevent coagulation, blood specimens to analyze immune cells and thrombocytes, presepsin, and PCT concentrations were kept with EDTA; analyzing D-dimers required obtaining the specimens from the citrate tubes. For detecting gasses and pH evaluation, tubes with heparin were used. Blood biochemistry was profiled using tubes with SiO2 to activate coagulation and get serum. All specimens underwent standard laboratory tests in a certified clinical laboratory. Blood gasses and acid–base balance were determined on an automatic analyzer (ABL815 Flex, Radiometer, Denmark) by the amperometric method using an oxygen galvanic cell with a solid electrolyte. Clinical blood analysis was performed on a hematological analyzer ADVIA 60 (Siemens Healthineers, Germany) by an integrated pack of TimePac reagent cartridges. Biochemical analyses were performed by the automatic biochemical analyzer Dirui CS-T240 and Dirui reagents (Dirui, China). IL-6 concentration in plasma was assessed using the Elecsys IL-6 reagent kit (Roche Diagnostics GmbH, Germany). The determination of D-dimers, C-reactive protein (CRP), presepsin, and procalcitonin (PCT) was performed using the immunochemiluminescent analyzer PATHFAST (LCI Medience Corporation, Japan).

### 2.3 Intervention

Patients who met the specified criteria underwent a hemoadsorption procedure with the Efferon CT device no later than 6 h after commencing MLV. The procedure known as hemadsorption was performed on the patient in two separate sessions, each lasting approximately 12 h, with an interval of 24 h between each treatment. The median overall duration of hemoadsorption for the patient was 23 h (IQR 19–25).

The Efferon CT adsorbent is a porous hypercrosslinked poly(styrene-co-divinylbenzene) (35). The retention of solutes takes place via adsorption on the surface and volumetric filling of micropores due to non-selective hydrophobic and *π*-π sorbent–sorbate interaction. The adsorber was rinsed with 1,000 mL of 0.9% saline solution with 5,000 IU of unfractionated heparin. Before hemoadsorption, patients were administered a bolus injection with 5,000 IU of unfractionated heparin. The bolus was repeated every 4 h during the period of each hemoadsorption. During hemoadsorption, heparin was injected into the extracorporeal circuit at a dose of 700–1,200 IU/h. The duration of the procedures was at least 12 h, using a standard dialysis catheter and a blood flow rate of 130–150 mL/min.

Hence, two sessions of hemadsorption with Efferon CT were performed in 65 patients with COVID-19. For eight patients, repeated hemadsorption lasted less than 6 h due to premature clotting of the extracorporeal circuit (three cases), bleeding from the tracheostomy area and catheter placement (three cases), catheter dislocation (one case), pneumothorax, and the need for surgical drainage of the pleural cavity (one case).

### 2.4 Data collection

The study included 130 patients on mechanical ventilation with a severe and critical course of COVID-19 from October 2020 to March 2022. The Efferon CT group included 65 patients, and data were collected prospectively and recorded in the research database. The control group of 65 patients receiving standard therapy was identified by hospital staff who were members of the research team through a retrospective review of paper medical charts conducted every 3 months. Cases with incomplete outcome data were excluded during the preliminary screening stage. The selection was performed according to a matched pair principle based on matching for at least five of six following factors: age, sex, common major comorbidity, SOFA scores at admission, vasopressor dose, and date of admission to the ICU (Figure 1). The data for the selected patients were transcribed from paper medical files into the research database. Two researchers conducted the data entry, performing cross-checks to ensure the accuracy of the collected information.

### 2.5 End points

The primary endpoint: “resolution of decompensated acute respiratory failure,” was calculated as the number of days without ventilatory support within 60 days of ventilator initiation (36) (a detailed definition is provided in the Supplementary materials on page 4). We also calculated the subdistribution hazard ratio (sHR) for the events “successful weaning from ventilator” and “death on ventilator.”

Secondary endpoints and scientific objectives are as follows: respiratory index (PaO_2_/FIO_2_), development of acute kidney injury, severity of MOD according to the SOFA scale, and number of days out of ICU within 60 days of MLV. The dynamics of clinical and laboratory parameters in the Efferon CT group were monitored at the beginning of MLV (point 0), in 2 and 12 h after the beginning of the first hemadsorption, and at the beginning and the end of the second hemoadsorption, as well as on days 3, 5, and 10 post-initiations of MLV. In the control group, laboratory and clinical parameters were measured at 1, 3, 5, and 10 days after starting the invasive MLV.

### 2.6 Statistical analysis

We used RStudio 2024 with R 4.3 for statistical analysis of the results. Data are presented as Me (Q1; Q3) or frequencies n/N (%). The Wilcoxon exact test was used for paired samples, and the Mann–Whitney U-test was used for unpaired samples. For binary variables, we used Fisher’s exact test for independent samples and calculated odds ratio (OR) with confidence interval (CI). McNemar’s test was used for repeated samples. Kaplan–Meier estimator and log-rank test were used to analyze the survival of the patients. The competing risk estimator and the Gray test were used for MLV- and ICU-stay outcomes. All results were considered statistically significant at a *p-value of* < 0.05. The values at non-matching time points were obtained by within-subject linear interpolation based on neighboring time points. The proportion of missing data in the study was less than 10% (3% in the Efferon CT group and 9% in the control group), and in such cases, interpolation based on neighboring time points was applied as described above; if interpolation was not feasible, the last observation carried forward (LOCF) method was used.

## 3 Results

### 3.1 Pre-treatment clinical parameters

The median age in the Efferon CT group was 61 years and in the control group was 63 years; there were 45 and 49% men, respectively, and the severity of organ dysfunction was SOFA Me = 4.0 points in both groups. The groups did not significantly differ in age, gender, SOFA score, body mass index, volume of lung lesions according to CT, or dose of vasoactive drugs needed to maintain hemodynamics (Table 1).

At the time of hospital admission, the SOFA values were Me (IQR): 1 (0; 1) in both groups (*p* = 0.964). During hospitalization, SOFA values increased by 2 and more scores in each patient included in the study due to the progression of the disease. The delta SOFA value was Me +3 (min: +2, max: +7) (*p* = 0.843) between groups. At the time of MLV initiation, both groups exhibited comparable SOFA values (Table 1), demonstrating the diagnosis of sepsis (viral) in each patient included in the study according to SEPSIS-3 criteria (37).

Prior to inclusion into the study, all patients received immunomodulating therapy with anti-IL-6 (olokizumab) or anti-IL-6 receptor (tocilizumab or levilimab) drugs or corticosteroids and were switched from non-invasive ventilation to invasive MLV. Patients exhibited no clinical and laboratory signs of sepsis or kidney stage 3 acute kidney injury (AKI), according to KDIGO (38), demonstrating no need for renal replacement therapy. No differences in major comorbidities between treatment groups were noticed (Supplementary Table S1).

Bacterial cultures grown from the sputum specimens harvested on the day of transfer of patients to the ICU showed no differences between study groups (Supplementary Table S2). Blood and urine cultures at the stage of inclusion in the study were negative in all patients.

### 3.2 Duration of mechanical ventilation and mortality during treatment of patients with COVID-19

The number of days without MLV during the 60-day study (MLV-free days) in the hemoadsorption group was significantly increased compared with the control group at the 90th percentile: 55 vs. 48 days, respectively (*p* < 0.0001, Table 2). Similar differences were revealed between groups in ICU-free days or any medical facility (*p* < 0.0001 and *p* < 0.007, respectively, Table 2). The cumulative incidence curves of the successful resolution of MLV and discharge from the ICU are shown in Supplementary Figure S1.

**Table 2:** Number of days without mechanical ventilation in ICU and healthcare facilities.

	Efferon CT group		Control group		
Parameter	Me (Q1; Q3)	*P90	Me (Q1; Q3)	*P90	**p-value
MLV-free days	40 (0; 52)	55	0 (0; 0)	48	<0.0001
ICU-free days	27 (0; 42)	47	0 (0; 0)	38	<0.0001
Hospital-free days	0 (0; 27)	35	0 (0; 0)	22	0.0070

Data are presented as Me (Q1; Q3). A detailed definition of “MLV-free days” is provided in the supplementary materials on page 4. *P90: 90th percentile. **Mann–Whitney U-test.

**Figure 2:**
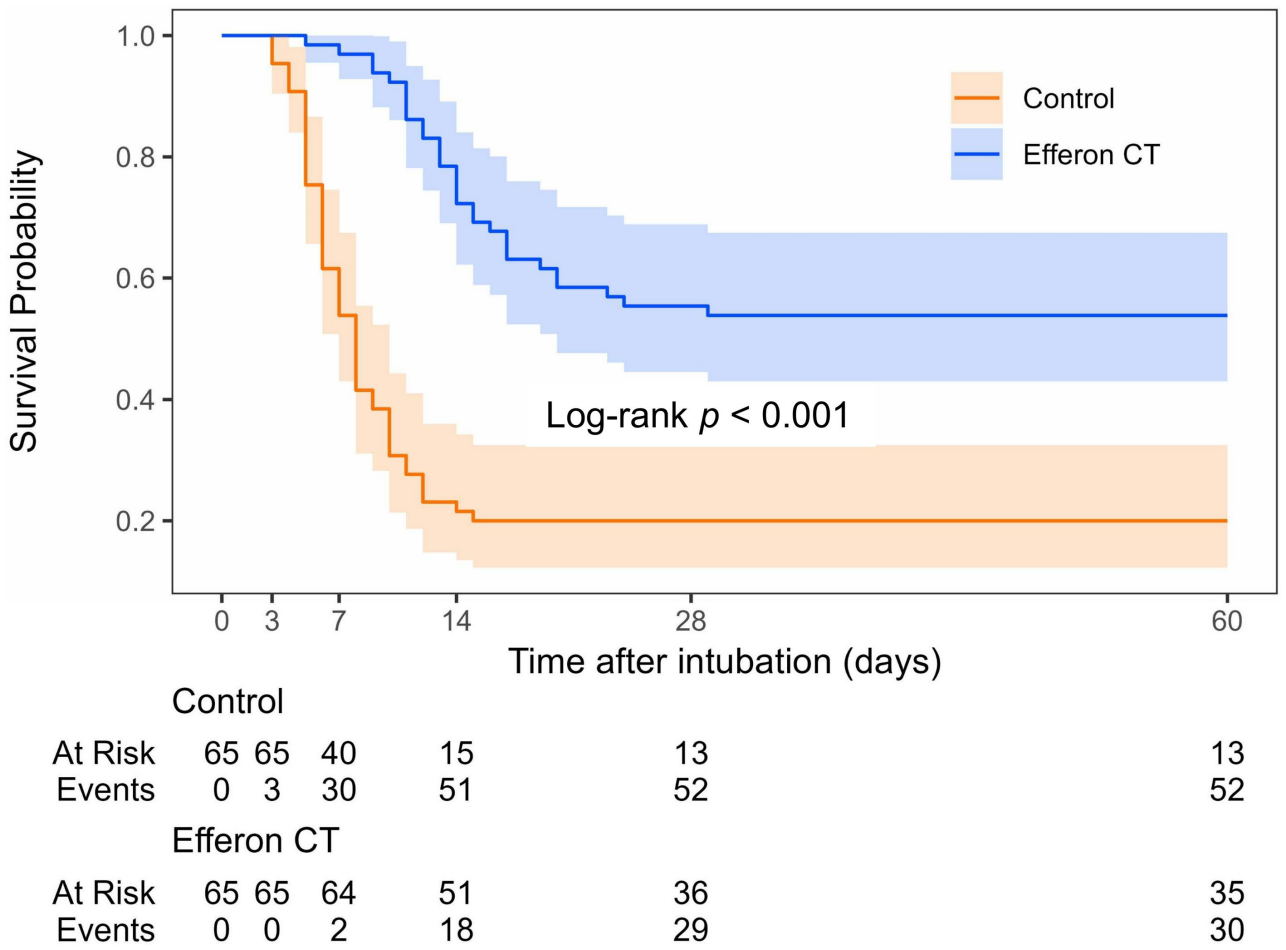
Kaplan–Meier survival curve for study group. Events correspond to deaths; 60-day survival: Control group–20% (13/65), Efferon CT group–54% (35/65).

As shown in Figure 2, starting from day 7 of inclusion into the study, mortality in the Efferon CT group had become significantly lower than in the control group (OR:0.04 (95% CI: 0.01–0.16), *p* < 0.001). In-hospital 28-day mortality and 60-day mortality were also lower in the Efferon CT group compared with the control group; OR values were, respectively, 0.20 (95% CI: 0.09–0.44; *p* < 0.001) and 0.21 (95% CI: 0.10–0.47; *p* < 0.001).

### 3.3 Changes in organ failure

At a baseline, there were no statistically or clinically significant differences between the Efferon CT group and the control group in the need for norepinephrine to maintain hemodynamics and in the indices of respiratory, renal, and MOF scores on the SOFA scale (Table 1).

Proportion of patients who required norepinephrine support to maintain hemodynamics was decreasing in hemoadsorption group compared to control group with significant differences on day 5 (14% vs. 37%, OR 0.26; 95%CI: 0.104–0.641, *p* = 0.003, Table 3).

**Table 3:** Dynamics of the need for norepinephrine and the development of AKI.

Variable	Group	Day 0	Day 3	Day 5	Day 10
Requirement for norepinephrine	Efferon CT	14/65 (21%)	13/65 (20%)	9/64* (14%)	6/60* (10%)
	Control	15/65 (23%)	17/62 (27%)	19/49* (38%)	1/20 (5%)
	*p*	1	0.439	0.003	0.820 (0.011)
Presence of signs of AKI (stages 1–2, KDIGO, 2012)	Efferon CT	5/65 (8%)	8/65 (12%)	9/64 (14%)	8/60 (13%)
	Control	4/65 (7%)	21/62** (34%)	18/49* (37%)	3/20 (15%)
	*p*	1	0.008	0.010	1 (0.020)

Data are presented as a fraction (%); the numerator is the number of patients requiring norepinephrine, and the denominator is the number of living patients at the time point in question.

* *p* < 0.05, ** *p* < 0.001 within-group McNemar’s test.

*p*—between-group chi-square test (*p*-value taking into account last observation carried forward, LOCF).

On day 5 in the Efferon CT group, the need for vasopressor support was observed only in 14% of patients and in the control group in 37% (OR 0.26; 95%CI: 0.104–0.641, *p* = 0.003, Table 3). Starting from day 3 post-extracorporeal treatment, the hemoadsorption significantly reduced the number of patients with signs of AKI (Table 3).

**Figure 3:**
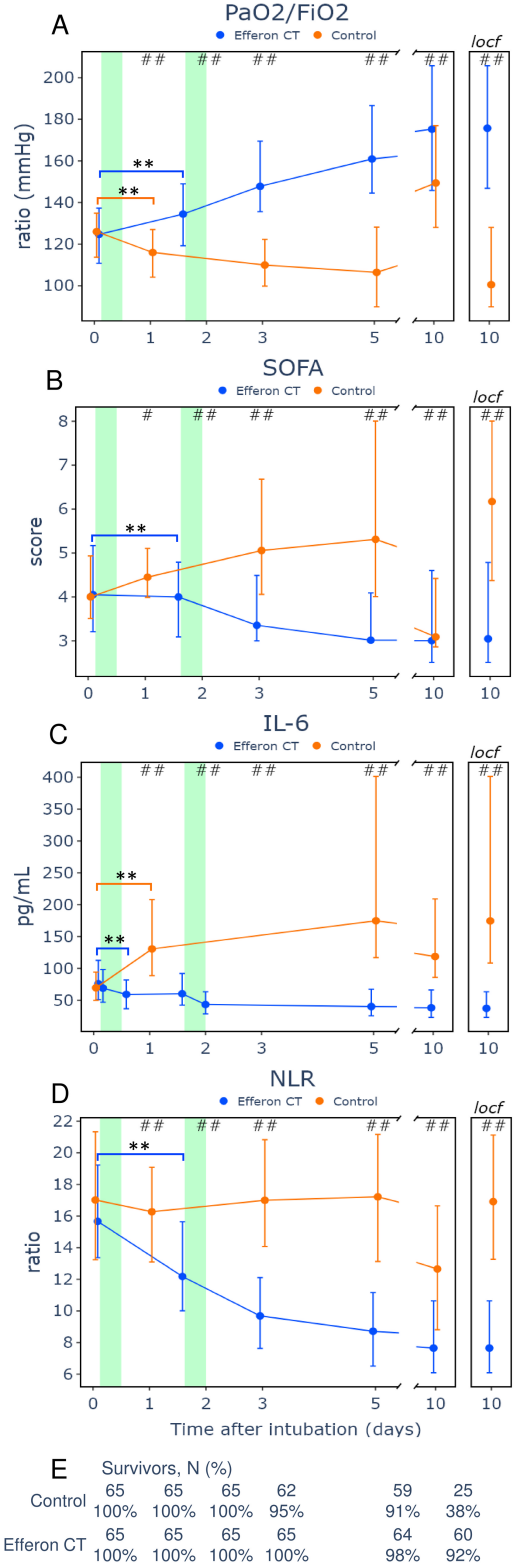
Dynamics of oxygenation indices **(A)**, multiple organ failure **(B)**, IL-6 **(С)**, neutrophil-to-lymphocyte ratio **(D)**, and numbers (%) of survived patients are shown under each day **(E)**. Data are presented as Me (IQR). # *p* < 0.05, ## *p* < 0.001 between-group Mann–Whitney U-test. * *p* < 0.05, ** *p* < 0.001 within-group Wilcoxon signed-rank test. LOCF–last observation carried forward.

Figure 3 presents an analysis in which deceased patients are included in the analyzed cohort using the LOCF method (last available observation carried forward to the day of death). In the control/standard treatment group, the oxygenation index consistently demonstrated a constant decline from 126 (116; 134) to 115 (104; 126) after 24 h (*p* < 0.0001) and to 109 (101; 119) after 3 days (*p* < 0.0001) (Figure 3A). In the control group, a significant increase of the oxygenation index, however, to a significantly lower value, 148 (128; 172), compared with the hemoadsorption group, was observed much later, on day 10 (Figure 3A). This increase was mainly due to the death of severe patients and their dropout from the analysis (Figure 3E), the so-called “survivor’s bias error,” which was further corroborated by the LOCF analysis presented in the figure.

In contrast to the control group, the oxygenation index of patients from the Efferon CT group after the first hemoadsorption increased at the 24-h point from 126 (113; 136) to 135 (120; 147), (*p* < 0.0001) (Figure 3A). Interestingly, in the Efferon CT group, the oxygenation index continued to increase after completion of the second hemadsorption, from 148 (136; 168) to 161 (146; 184) on day 5 (*p* = 0.0005). The opposite dynamics of the oxygenation index in the Efferon group vs. the control group exhibited statistically significant differences at all study points (Figure 3A).

Analysis of the SOFA scale dynamics revealed that in the Efferon CT group, the severity of MOD significantly and steadily decreased after the first hemoadsorption to day 5, with no further changes on day 10. In contrast, in the control group, SOFA scale values significantly and gradually increased from day 0 to day 5 (Figure 3B). Importantly, the lactate dynamics (Supplementary Figure S2A) followed in the same direction as SOFA dynamics and in the opposite direction to indeх oxygenation changes linking hypoperfusion or respiratory failure with intensity of anaerobic glycolysis in tissues and MOD development in COVID-19 sepsis.

### 3.4 Dynamics of pro-inflammatory markers during treatment

Elevated IL-6 levels contribute to the pathogenesis and severity of COVID-19, which is associated with the prolongation of hospital stay and earlier mortality and increased mortality of COVID-19 patients (39). Figure 3C illustrates that, prior to hemoadsorption, the hemoadsorption group did not differ from the control group on day 0 in the cytokine level. However, hemoadsorption treatment induced a significant decrease in IL-6 levels in plasma, whereas in the control group, the values of IL-6 remained constantly increased (Figure 3C). Between-group differences became significant in 24 h (*p* = 0.0002), remaining significantly higher on day 5 in the hemoadsorption group than in the control group (Figure 3C).

Increased ferritin concentrations associated with COVID-19 severity contribute to the immunopathogenesis of the disease (40, 41). In our study, initial high levels of ferritin rapidly, during the first day, declined only in the hemoadsorption group (from 767 (542; 964) to 533 (432; 660) ng/ml, *p* < 0.0001, Supplementary Figure S2B). In contrast, the control group consistently elevated ferritin levels throughout the observation period. The between-group difference was statistically significant during the 10-day monitoring (*p* < 0.0001) at any time point (Supplementary Figure S2B).

Another classic biomarker of inflammation is the level of leukocytes. The hemoadsorption induced a decrease in the content of well-known biomarkers of the systemic inflammatory response, and the levels of IL-6 and ferritin in the plasma of patients corresponded to a decrease in the content of leukocytes in the blood. Supplementary Figure S2C shows that the reduction in leukocyte concentration vs. time point 0 was significant starting from the 36th h onward during hemoadsorption, in contrast to the control group, where leukocyte content rapidly increased despite standard treatment and remained enhanced at least until the 10th day. Significant between-group differences were observed at all time points of the study (Supplementary Figure S2C).

The ratio of immune cells (neutrophils/lymphocytes ratio, NLR) in the control group remained unchanged over 5 days, amounting to 16.8 (13.3; 21.8) at a time point 0 and 16.6 (13.3; 21.06) at day 5 (*p* = 0.704) (Figure 3D). The drop in NLR value on the 10th day in the control group was associated with the survivor’s bias because the values did not decrease, considering LOCF (Figure 3D). In contrast to the control group, the hemoadsorption group exhibited a steady, early-onset decrease in the NLR values: from a value of 15.6 (13.4; 19.06) at a time point 0 to 12.2 (10.3; 15.6) after the first hemoadsorption at a time point 36 h (*p* < 0.0001) and up to 8.4 (6.3; 10.7) on day 5 (*p* < 0.0001) (Figure 3D). Between-group differences were significant at all time points throughout the study.

Interestingly, the dynamics of adaptive immunity and innate immunity cells, lymphocytes, and neutrophils behaved in a contrasting fashion depending on the treatment: Standard treatment provided an increased innate immunity (neutrophil count) trend during the first 10 days of observation, while the adaptive immunity (lymphocyte changes) remained decreased. Hemoadsorption, and vice versa, yielded a trend with the increasing number of lymphocytes, while the neutrophil count trend remained steady and decreased during the 5-day and 10-day observations (Supplementary Figures S2D,E).

A comparison of the clinical and laboratory parameters of patients with COVID-19 during treatment revealed that patients treated with hemoadsorption exhibited more favorable dynamics by many quantitative molecular/biochemical biomarkers (procalcitonin, presepsin, lactate, glucose, creatinine, platelets, liver enzymes and bilirubin, creatine phosphokinase, and lactate dehydrogenase) and platelets values in comparison with the control (Table 4).

**Table 4:** Comparative dynamics of clinical and laboratory parameters of patients with COVID-19 during treatment.

Variable	Group	Baseline	24 h	3rd day	5th day
Procalcitonin, ng/mL	Efferon CT	0.16 (0.12; 0.19)	0.14 (0.12; 0.21)**	0.16 (0.12; 0.23)	0.16 (0.14; 0.27)
	Control	0.16 (0.12; 0.22)	0.17 (0.14; 0.29)**	0.19 (0.16; 0.42)**	0.26 (0.18; 0.67)**
	*p*-value	0.594	0.058	<0.001	<0.001
Presepsin, pg./mL	Efferon CT	392 (338; 430)	373 (343; 418)**	379 (351; 420)	387 (359; 457)
	Control	391 (347; 452)	423 (374; 506)**	488 (406; 712)**	486 (418; 729)**
	*p*-value	0.73	0.003	<0.001	<0.001
CRP, mg/L	Efferon CT	7.6 (5.5; 10.5)	6.4 (5.1; 9.7)**	5.5 (4.1; 7.2)**	5.6 (4.6; 7.4)**
	Control	7.9 (5.2; 13.4)	7.3 (5.7; 10.2)	7.6 (5.9; 10.7)	8.4 (6.5; 12.1)*
	*p*-value	0.637	0.184	<0.001	<0.001
Lactate, mmol/L	Efferon CT	4.3 (3.9; 5.3)	4.3 (3.6; 5.05)*	3.8 (3.1; 4.5)**	3.6 (2.9; 4.4)**
	Control	4.5 (3.8; 5.3)	4.7 (4.1; 5.4)*	4.9 (4.3; 5.6)**	4.9 (4.4; 5.9)**
	*p*-value	0.831	0.034	<0.001	<0.001
Glucose, mmol/L	Efferon CT	9.9 (8.9; 11.5)	9 (8.0; 10.05)**	9 (7.6; 10.4)*	8.9 (7.4; 10.3)*
	Control	9.6 (8.5; 11.3)	10.5 (9.3; 13.2)**	11.4 (10.3; 13.3)**	12.2 (10.2; 14.2)**
	*p*-value	0.441	<0.001	<0.001	<0.001
Creatinine, μmol/L	Efferon CT	97 (89; 109)	93 (84; 105)	95 (87; 105)	97 (85; 111)
	Control	94 (84; 108)	102 (92; 124)**	117 (99; 140)**	116 (103; 159)**
	*p*-value	0.322	0.001	<0.001	<0.001
Platelets, x10^9^/L	Efferon CT	265 (217; 300)	253 (209; 297)**	242 (205; 284)**	240 (204; 285)*
	Control	248 (196; 308)	222 (189; 285)**	195 (153; 240)**	176 (147; 220)**
	p-value	0.646	0.097	<0.001	<0.001
aPTT, sec	Efferon CT	28 (26; 30)	31 (28; 35)**	34 (31; 37)**	35 (32; 38)**
	Control	29 (26; 31)	28 (27; 30)	29 (27; 32)*	29 (28; 32)**
	*p*-value	0.834	<0.001	<0.001	<0.001
ALAT, units/L	Efferon CT	51 (37; 76)	52 (37; 83)**	41 (35; 59)**	39 (33; 52)**
	Control	59 (37; 84)	55 (41; 89)**	69 (45; 95)**	74 (54; 98)**
	*p*-value	0.71	0.199	<0.001	<0.001
Amylase, units/L	Efferon CT	72 (59; 87)	68 (57; 81)	70 (57; 86)	74 (62; 86)
	Control	68 (54; 86)	82 (72; 103)**	95 (80; 142)**	102 (85; 138)**
	*p*-value	0.69	<0.001	<0.001	<0.001
Total bilirubin, μmol/L	Efferon CT	12 (9; 16)	11 (9; 14)**	10 (8; 12)**	10 (8; 12)**
	Control	12 (9; 16)	13 (9; 15)	12 (10; 16)	13 (11; 16)
	*p*-value	0.463	0.075	<0.001	<0.001
CPK, ng/mL	Efferon CT	158 (127; 217)	147 (106; 191)*	136 (99; 193)*	129 (96; 178)*
	Control	147 (107; 218)	168 (137; 285)*	169 (138; 310)**	175 (141; 257)**
	*p*-value	0.178	0.017	0.002	<0.001
LDH, units/L	Efferon CT	1,319 (1,007; 1,653)	1,189 (1,019; 1,420)**	1,080 (932; 1,210)**	1,042 (922; 1,195)**
	Control	1,268 (1,053; 1,503)	1,410 (1,107; 1885)**	1,437 (1,122; 1867)**	1,459 (1,193; 1920)**
	*p*-value	0.587	0.002	<0.001	<0.001

*p < 0.05, **p < 0.001: within-group comparison of a biomarker against day 0, paired Wilcoxon test. *p*-value: between-group Mann–Whitney U-test (Efferon CT vs. control).

CRP, C-reactive protein; aPTT, activated partial thromboplastin time; ALAT, alanine aminotransferase; CPK, creatine phosphokinase; LDH, lactate dehydrogenase.

Of the 130 hemoadsorption procedures performed, only 8 (6%) were terminated prematurely. These cases were due to column thrombosis (three cases), bleeding from the tracheostomy area and catheter placement (three cases), catheter dislocation (one case), pneumothorax, and the need for surgical drainage of the pleural cavity (one case). Data confirm the safety of hemoadsorption using the Efferon CT in patients with COVID-19.

## 4 Discussion

This observational study revealed that hemadsorption using the Efferon CT sorbent led to rapid and significant clinically beneficial changes, including a reduction in the necessity for norepinephrine, an increase in the oxygenation index, prevention of the sepsis-associated AKI, a decrease in the development of MOD and immune system indicators, including the markers of innate immunity and adaptive immunity and their balance, NLR, as well as pro-inflammatory IL-6 and ferritin levels.

It is important to highlight the efficacy of hemoadsorption using Efferon CT, which led to the resolution of acute respiratory failure in 54% of patients (Supplementary Figure S1A). At the same time, the number of days without mechanical ventilation significantly increased (Table 2). The index of oxygenation exhibited an early increase from the first day after the invasive MLV initiation, in comparison with the control group, where a persistent decline was evident over the initial 5-day period of treatment.

Previous studies utilizing CytoSorb cartridges for hemoadsorption have demonstrated that the prevention or deceleration of the deterioration of renal and respiratory function is a significant factor in evaluating the therapeutic efficacy of hemoadsorption in a critical illness patient, including that of COVID-19 (42). During the development of the pandemic, the utilization of hemoadsorption technologies for the treatment of COVID-19 underwent a number of stages.

The initial stage of evaluating the feasibility of removing pro-inflammatory cytokines and other unwanted, including toxic, PAMP/DAMP molecules with a molecular weight of up to 60 kDa from the body occurred very soon after the understanding of the absence of effective etiotropic antiviral therapy because of the extremely high mortality rates due to failure of traditional prosthetics for impaired vital functions (43).

In April 2020, the US Food and Drug Administration (FDA) approved hemoadsorption in emergency cases requiring the removal of inflammatory mediators, including cytokines, from the body. The employment of such a therapy has been shown to contribute to the reduction of hyperinflammation, world-widely recognized as a critical component pathogenetically linked to the development of life-threatening, severe, and critical courses of COVID-19 (12, 44).

Our study revealed several new aspects of the use of hemoadsorption in patients with COVID-19. First, the study demonstrated the potential for hemoadsorption procedures to produce various clinically significant effects associated with rapid reconstitution of pathogenetically linked biomarkers in patients with severe and extremely severe (critical) COVID-19, who require urgent MLV for life support. Second, our data directly demonstrate that the rapid progression of MOD, a feature of COVID-19 patients with severe and critical forms of the disease, was interrupted when Efferon CT cartridges were employed. Third, the significant effect of hemoadsorption on mortality in our setting shows the promise for clinical use in severe and critical viral sepsis, including septic shock patients. Fourth, our study demonstrated the potential of Efferon CT-mediated hemoadsorption to restore the balance of the adaptive immune system and innate immune system impaired in COVID-19. Indeed, the biomarker of this disbalance, the NLR value, significantly increased post-hemoadsorption due to lymphocyte and neutrophil counts increasing and decreasing, respectively.

We suggest that the hemoadsorption is beneficial for patients with severe and critical course of the disease because of starting hemoadsorption relatively early, within the first 6 h following the commencement of invasive MLV, prior to the development of MLV-induced lung injury. The latter may further promote adaptive immunity/innate immunity disbalance that pathogenetically contributes to the DAMP/PAMP storm. The early initiation of hemoadsorption treatment may result in early detoxification of DAMP/PAMP factors, providing a “temporary niche” to restore the immune system and lung tissue functions prior to the build-up of irreversible damage to adaptive immunity system, lung parenchyma, and tissues of other organs. The “permanent niche” with decreased concentration of circulating DAMP/PAMP may prevent living cells from insufficient oxygen perfusion and excessive pro-oxidant and pro-inflammatory activity of innate immune cells, presumably due to inducing mechanisms of cryoprotection to further alterations of vascular endothelium and organ parenchyma.

It is notable that the dynamics of cumulative weaning increase from MLV corresponds to dynamics of cumulative survival rate in both the Efferon CT group and control group, wherein the favorable effect of hemoadsorption is evident for both parameters in both groups (Figure 2). This highlights the significance of the development of acute respiratory failure as the primary cause of mortality in cases of severe and critical COVID-19 course and the necessity to prevent further deterioration of acute respiratory failure and halt the progression of MOF to enhance survival. The timely use of adjuvant pathogenetically based therapeutic treatment, including hemoadsorption, as a method of active immunomodulation, is intended to become one of the promising life support strategies for patients suffering from severe and critical forms of COVID-19 as a life-threatening viral sepsis.

This study has potential limitations. First, the estimated effects of hemoadsorption were observed in a prospective cohort study, whereas the control cohort was based on a retrospectively matched patients’ cohort instead of randomization of patients. This design might lead to selection bias. However, the control group matching resulted in no statistically significant differences in key demographic and clinical parameters between groups that may potentially affect the bias. Second, because of the number of patients under the hemoadsorption treatment (*n* = 65), the study seems underpowered to reveal and validate candidate biomarkers for more personalized treatment of severe and critical COVID-19 patients with hemoadsorption. Third, patients were enrolled at a single center; the absence of a validation cohort from another hospital, including patients from different geographical region(s), constrained the generalizability of the findings. Fourth, the study did not investigate the influence of viral load or the roles of different virus variants on the results obtained. Instead, the presumable estimation was based on published epidemiological data from other hospitals of the same region, enrollment rate similarities, and limited data (Supplementary Table S3). Finally, since COVID-19 remains an ongoing global health challenge with continuous virus mutations and the emergence of new virus strains, this factor should also be considered a study limitation.

Hence, our data demonstrate that the use of hemoadsorption employing the Efferon CT cartridge is a safe and effective treatment procedure that successfully resolves acute respiratory failure in 54% of patients with severe and critical forms of COVID-19. The number of MLV-free and ICU-free days in the Efferon CT group was increased on 7 days and 9 days, respectively, vs. the control group. From the first day after the commencement of MLV in the hemoadsorption group, a significant increase in the oxygenation index was observed from day 1 to day 5 compared to the control group patients. The number of patients with AKI stages 1–2 (KDIGO, 2012) on day 5 was statistically significantly lower in the Efferon CT group compared to the control group. Furthermore, patients in the hemoadsorption group on day 5 of the study required significantly fewer vasoactive and inotropic drugs and exhibited lower MOF values than patients in the control group.

Hemoadsorption resulted in a statistically significant reduction in IL-6, ferritin, procalcitonin, and presepsin, increasing the lymphocyte count and decreasing neutrophil/lymphocyte ratio, lactate, and glucose values vs. the control group. Data suggest that improvements in pathogenetically relevant clinical parameters and serum markers of inflammation along with adaptive immunity/innate immunity balance restoration following hemoadsorption with Efferon CT cartridges contributed to survival benefit of patients by decreasing the 60-day mortality from 80 to 46% vs. matched control group.

## Data Availability

The raw data supporting the conclusions of this article will be made available by the authors, without undue reservation.

## References

[1] National Institutes of Health (2021) COVID-19 treatment guidelines panel. coronavirus disease 2019 (COVID-19) treatment guidelines. Available at: . (https://www.covid19treatmentguidelines.nih.gov/) 34003615

[2] Bolker A Coe K Smith J Stevenson K Wang S-H Reed E. Predictors of respiratory bacterial co-infection in hospitalized COVID-19 patients. Diagn Microbiol Infect Dis. (2022) 102:115558. doi: 10.1016/j.diagmicrobio.2021.115558, PMID: 34731685 PMC8481625

[3] Wu C Chen X Cai Y Xia J Zhou X Xu S. Risk factors associated with acute respiratory distress syndrome and death in patients with coronavirus disease 2019 pneumonia in Wuhan, China. JAMA Intern Med. (2020) 180:934–43. doi: 10.1001/jamainternmed.2020.0994, PMID: 32167524 PMC7070509

[4] Armstrong RA Kane AD Cook TM. Outcomes from intensive care in patients with COVID-19: a systematic review and meta-analysis of observational studies. Anaesthesia. (2020) 75:1340–9. doi: 10.1111/anae.15201 32602561

[5] Malaeb R Haider A Abdulateef M Hameed M Daniel U Kabilwa G. High mortality rates among COVID-19 intensive care patients in Iraq: insights from a retrospective cohort study at Médecins Sans Frontières supported hospital in Baghdad. Front Public Health. (2023) 11:1185330. doi: 10.3389/fpubh.2023.1185330, PMID: 37719728 PMC10501727

[6] Andreev SS Ketskalo MV Narusova PO Lysenko MA. Secondary infections in patients with extremely severe COVID-19 during ECMO therapy. Gen Reanimat. (2023) 19:4–13. doi: 10.15360/1813-9779-2023-2-2265

[7] Ronco C Reis T. Kidney involvement in COVID-19 and rationale for extracorporeal therapies. Nat Rev Nephrol. (2020) 16:308–10. doi: 10.1038/s41581-020-0284-7, PMID: 32273593 PMC7144544

[8] Xu Z Shi L Wang Y Zhang J Huang L Zhang C. Pathological findings of COVID-19 associated with acute respiratory distress syndrome. Lancet Respir Med. (2020) 8:420–2. doi: 10.1016/S2213-2600(20)30076-X, PMID: 32085846 PMC7164771

[9] Ruiz-Rodríguez JC Plata-Menchaca EP Chiscano-Camón L Ruiz-Sanmartin A Ferrer R. Blood purification in sepsis and COVID-19: what’s new in cytokine and endotoxin hemoadsorption. J Anesth Analg Crit Care. (2022) 2:15. doi: 10.1186/s44158-022-00043-w, PMID: 37386575 PMC8978509

[10] Wolley M Jardine M Hutchison CA. Exploring the clinical relevance of providing increased removal of large middle molecules. Clin J Am Soc Nephrol. (2018) 13:805–14. doi: 10.2215/CJN.10110917, PMID: 29507008 PMC5969479

[11] Kuwana T Kinoshita K Ihara S Sawada N Hosokawa T Mutoh T. The characteristics of patients with severe COVID-19 pneumonia treated with direct Hemoperfusion using Polymyxin B-immobilized Fiber column (PMX-DHP). Infect Drug Resist. (2022) 15:4819–28. doi: 10.2147/IDR.S374920, PMID: 36043160 PMC9420440

[12] Chang K Li Y Qin Z Zhang Z Wang L Yang Q. Effect of extracorporeal hemoadsorption in critically ill patients with COVID-19: a narrative review. Front Immunol. (2023) 14:1074465. doi: 10.3389/fimmu.2023.1074465, PMID: 36817416 PMC9936071

[13] Alavi Darazam I Kazempour M Pourhoseingholi MA Hatami F Rabiei MM Javandoust Gharehbagh F. Efficacy of Hemoperfusion in severe and critical cases of COVID-19. Blood Purif. (2023) 52:8–16. doi: 10.1159/000524606, PMID: 35580567 PMC9393767

[14] Yakubtsevich RE Rakashevich DN. Hemoadsorption in patients with various types of respiratory support for severe COVID-19. Gen Reanimat. (2022) 18:10–7. doi: 10.15360/1813-9779-2022-5-10-17

[15] Mitzner S Kogelmann K Ince C Molnár Z Ferrer R Nierhaus A. Adjunctive Hemoadsorption therapy with CytoSorb in patients with septic/Vasoplegic shock: a best practice consensus statement. J Clin Med. (2023) 12:7199. doi: 10.3390/jcm12237199, PMID: 38068250 PMC10707447

[16] Stahl K David S. Introductory editorial for the thematic collection “blood purification in sepsis: from bench to bedside” in intensive care medicine experimental. Intensive Care Med Exp. (2023) 11:68. doi: 10.1186/s40635-023-00555-x, PMID: 37777664 PMC10542035

[17] Stahl K Bode C Seeliger B Wendel-Garcia PD David S. Current clinical practice in using adjunctive extracorporeal blood purification in sepsis and septic shock: results from the ESICM “EXPLORATION” survey. Intensive Care Med Exp. (2024) 12:5. doi: 10.1186/s40635-023-00592-6, PMID: 38238627 PMC10796869

[18] Niazi NS Nassar TI Stewart IJ Honore PM Sharma K Chung KK. A review of extracorporeal blood purification techniques for the treatment of critically ill coronavirus disease 2019 patients. ASAIO J. (2022) 68:1219–27. doi: 10.1097/MAT.0000000000001761, PMID: 35417433 PMC9521577

[19] Ronco C Bellomo R. Hemoperfusion: technical aspects and state of the art. Crit Care. (2022) 26:135. doi: 10.1186/s13054-022-04009-w, PMID: 35549999 PMC9097563

[20] Ricci Z Romagnoli S Reis T Bellomo R Ronco C. Hemoperfusion in the intensive care unit. Intensive Care Med. (2022) 48:1397–408. doi: 10.1007/s00134-022-06810-1, PMID: 35984473 PMC9389493

[21] De Rosa S Marengo M Fiorentino M Fanelli V Brienza N Fiaccadori E. Extracorporeal blood purification therapies for sepsis-associated acute kidney injury in critically ill patients: expert opinion from the SIAARTI-SIN joint commission. J Nephrol. (2023) 36:1731–42. doi: 10.1007/s40620-023-01637-5, PMID: 37439963 PMC10543830

[22] Kovzel VA Davydova LA Karzin AV Tsarenko SV Baturova VY Polupan AA. Methods of extracorporeal Hemocorrection in Sepsis (review). Gen Reanimat. (2023) 19:68–82. doi: 10.15360/1813-9779-2023-2-2282

[23] Yavuz T Orhan S Rollas K Toksoy CK Kazan ED Bozkurt E. Evaluation of clinical features and laboratory findings in critical intensive care unit patients with severe coronavirus disease-19 who underwent extracorporeal cytokine adsorption. Ther Apher Dial. (2023) 27:890–7. doi: 10.1111/1744-9987.14001, PMID: 37177852

[24] Supady A Weber E Rieder M Lother A Niklaus T Zahn T. Cytokine adsorption in patients with severe COVID-19 pneumonia requiring extracorporeal membrane oxygenation (CYCOV): a single Centre, open-label, randomised, controlled trial. Lancet Respir Med. (2021) 9:755–62. doi: 10.1016/S2213-2600(21)00177-6, PMID: 34000236 PMC8121541

[25] Koc S Uysal H. Literature review of Hemadsorption therapy in severe COVID-19 cases: a narrative review. Clin Lab. (2022) 68:839. doi: 10.7754/Clin.Lab.2021.210839, PMID: 35142202

[26] Hayanga JWA Song T Durham L Garrison L Smith D Molnar Z. Extracorporeal hemoadsorption in critically ill COVID-19 patients on VV ECMO: the CytoSorb therapy in COVID-19 (CTC) registry. Crit Care. (2023) 27:243. doi: 10.1186/s13054-023-04517-3, PMID: 37337243 PMC10280833

[27] Kusirisin P Noppakun K Trongtrakul K Vongsanim S Suteeka Y Ophascharoensuk V. Efficacy of the cytokine adsorption therapy in patients with severe COVID-19-associated pneumonia: lesson learned from a prospective observational study. Blood Purif. (2024) 53:10–22. doi: 10.1159/000534914, PMID: 37918373 PMC11251652

[28] Masolitin SV Protsenko DN Tyurin IN Magomedov MA Kim TG Grishina LA. The early use of selective Hemoadsorption based on a hyper-crosslinked styrene-Divinylbenzene copolymer in patients with toxic rhabdomyolysis complicated by acute kidney injury (multicenter randomized clinical trial). Gen Resuscit. (2022) 18:22–9. doi: 10.15360/1813-9779-2022-6-22-29

[29] Ratnikov VA Sheglov AN Abramovskiy SV Simutis IS Danilov MS Ivanova GG. Predictors of clinical efficacy of cytokine Hemoadsorption in COVID-19 (clinical trial). Gen Reanimat. (2023) 19:20–6. doi: 10.15360/1813-9779-2023-1-2224

[30] Rey S Kulabukhov VM Popov A Nikitina O Berdnikov G Magomedov M. Hemoperfusion using the lps-selective mesoporous polymeric adsorbent in septic shock: a multicenter randomized clinical trial. Shock. (2023) 59:846–54. doi: 10.1097/SHK.0000000000002121, PMID: 37018802 PMC10227945

[31] Pisarev VM Rey SI Kulabukhov VV Popov AY. If EAA test versus LAL test is always clinically better and whether any test always relates to the therapeutic value of Hemoperfusion in septic shock patients? Shock. (2023) 60:725–6. doi: 10.1097/SHK.0000000000002201, PMID: 37549019

[32] Eremenko AA Marchenko TV Nikoda VV Zokoev AK Skripalenko DA. Endotoxin and cytokines removal with adsorption device in a child with Sepsis after Transplantectomy (case report). Gen Reanimat. (2023) 19:48–53. doi: 10.15360/1813-9779-2023-6-48-53

[33] Stepanenko SM Afukov II Aleksandrovich YS Zilbert EV Chatchukhina AB Turischev IV. P220 Hemoperfusion with Efferon LPS NEO device reverts multiple organ failure, decreases IL-6 and TNF levels and systemic immune inflammation in children with sepsis. Crit Care. (2024) 28:68. doi: 10.1186/s13054-024-04822-5

[34] The World Medical Association. (2024). WMA Declaration of Helsinki – Ethical principles for medical research involving human participants. Available at: (https://www.wma.net/policies-post/wma-declaration-of-helsinki/) 10.1001/jama.2024.21972 39425955

[35] Davankov VA Tsyurupa MP. Hypercrosslinked polymeric networks and adsorbing materials: synthesis, properties, structure, and applications. New York, NY: Elsevier (2010).

[36] Yehya N Harhay MO Curley MAQ Schoenfeld DA Reeder RW. Reappraisal of ventilator-free days in critical care research. Am J Respir Crit Care Med. (2019) 200:828–36. doi: 10.1164/rccm.201810-2050CP, PMID: 31034248 PMC6812447

[37] Singer M Deutschman CS Seymour CW Shankar-Hari M Annane D Bauer M. The third international consensus definitions for Sepsis and septic shock (Sepsis-3). JAMA. (2016) 315:801–10. doi: 10.1001/jama.2016.0287, PMID: 26903338 PMC4968574

[38] Khwaja A. KDIGO clinical practice guidelines for acute kidney injury. Nephron Clin Pract. (2012) 120:c179–84. doi: 10.1159/000339789, PMID: 22890468

[39] Nikkhoo B Mohammadi M Hasani S Sigari N Borhani A Ramezani C. Elevated interleukin (IL)-6 as a predictor of disease severity among Covid-19 patients: a prospective cohort study. BMC Infect Dis. (2023) 23:311. doi: 10.1186/s12879-023-08294-w, PMID: 37161412 PMC10169099

[40] Ramos Rojas MC Cuaresma Cuadros EA Cayo Castillo JJ Monasterio Benique DA. Association of biomarkers and severity of COVID-19: a crosssectional study. Medwave. (2022) 22:e002548. doi: 10.5867/medwave.2022.06.002548 35917254

[41] Volfovitch Y Tsur AM Gurevitch M Novick D Rabinowitz R Mandel M. The intercorrelations between blood levels of ferritin, sCD163, and IL-18 in COVID-19 patients and their association to prognosis. Immunol Res. (2022) 70:817–28. doi: 10.1007/s12026-022-09312-w, PMID: 36222965 PMC9555272

[42] Alharthy A Faqihi F Memish ZA Balhamar A Nasim N Shahzad A. Continuous renal replacement therapy with the addition of CytoSorb cartridge in critically ill patients with COVID-19 plus acute kidney injury: a case-series. Artif Organs. (2021) 45:E101–12. doi: 10.1111/aor.13864, PMID: 33190288 PMC7753655

[43] Ronco C Navalesi P Vincent JL. Coronavirus epidemic: preparing for extracorporeal organ support in intensive care. Lancet Respir Med. (2020) 8:240–1. doi: 10.1016/S2213-2600(20)30060-6, PMID: 32035509 PMC7154507

[44] Moore JB June CH. Cytokine release syndrome in severe COVID-19. Science. (2020) 368:473–4. doi: 10.1126/science.abb8925 32303591

