# Supplementary material for: Efficacy of hemoadsorption in the severe course of COVID-19
Source: Front Med (Lausanne). 2025 Mar 6;12:1491137. doi: 10.3389/fmed.2025.1491137 (PMC11922909; doi:10.3389/fmed.2025.1491137)
Supplement: Supplementary file 1 [file Data_Sheet_1.docx]

**Supplementary Materials**


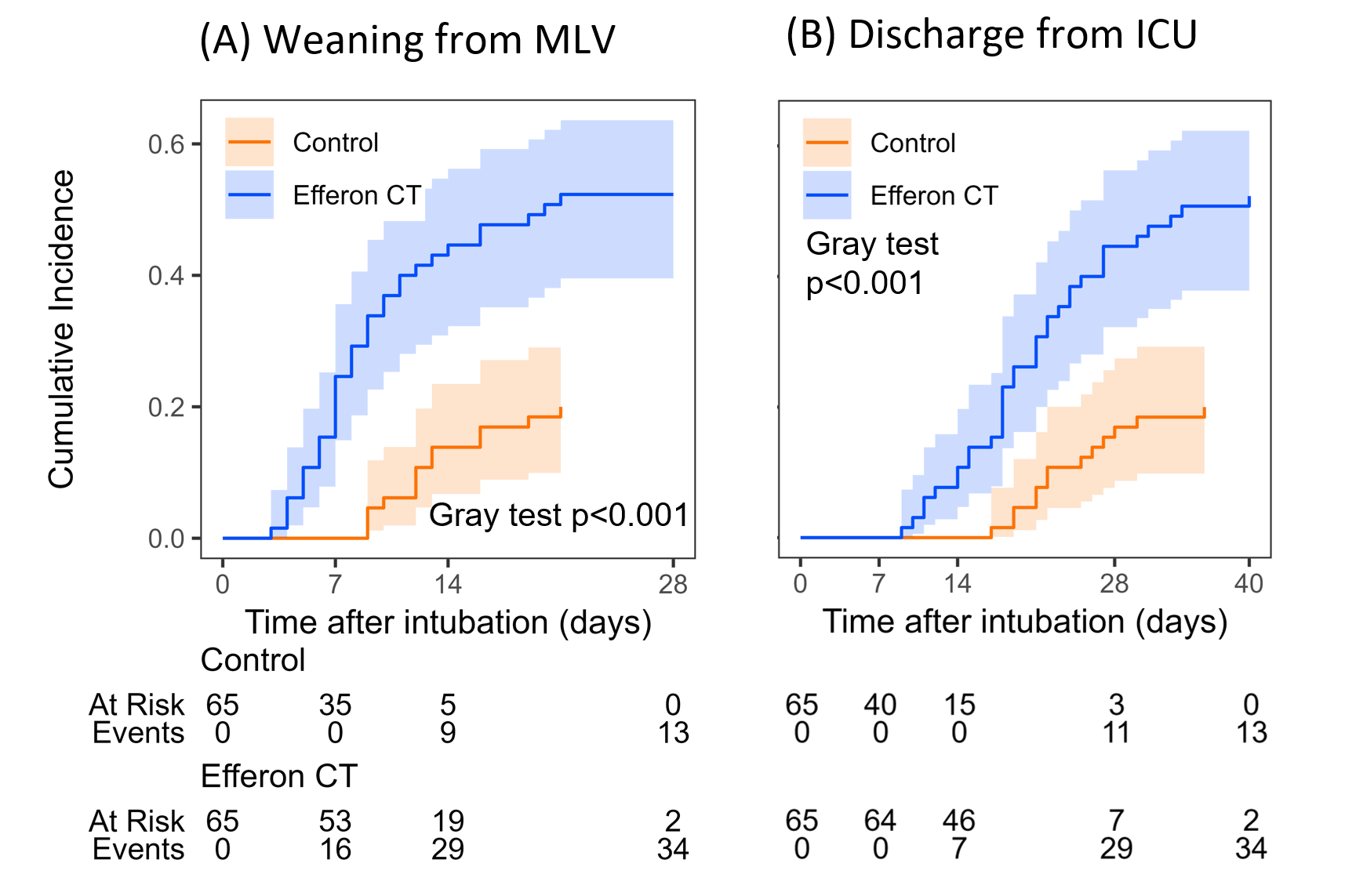


**Figure S1.** Cumulative incident curves corresponding to: successful weaning from MLV (A), discharge from the ICU (B).


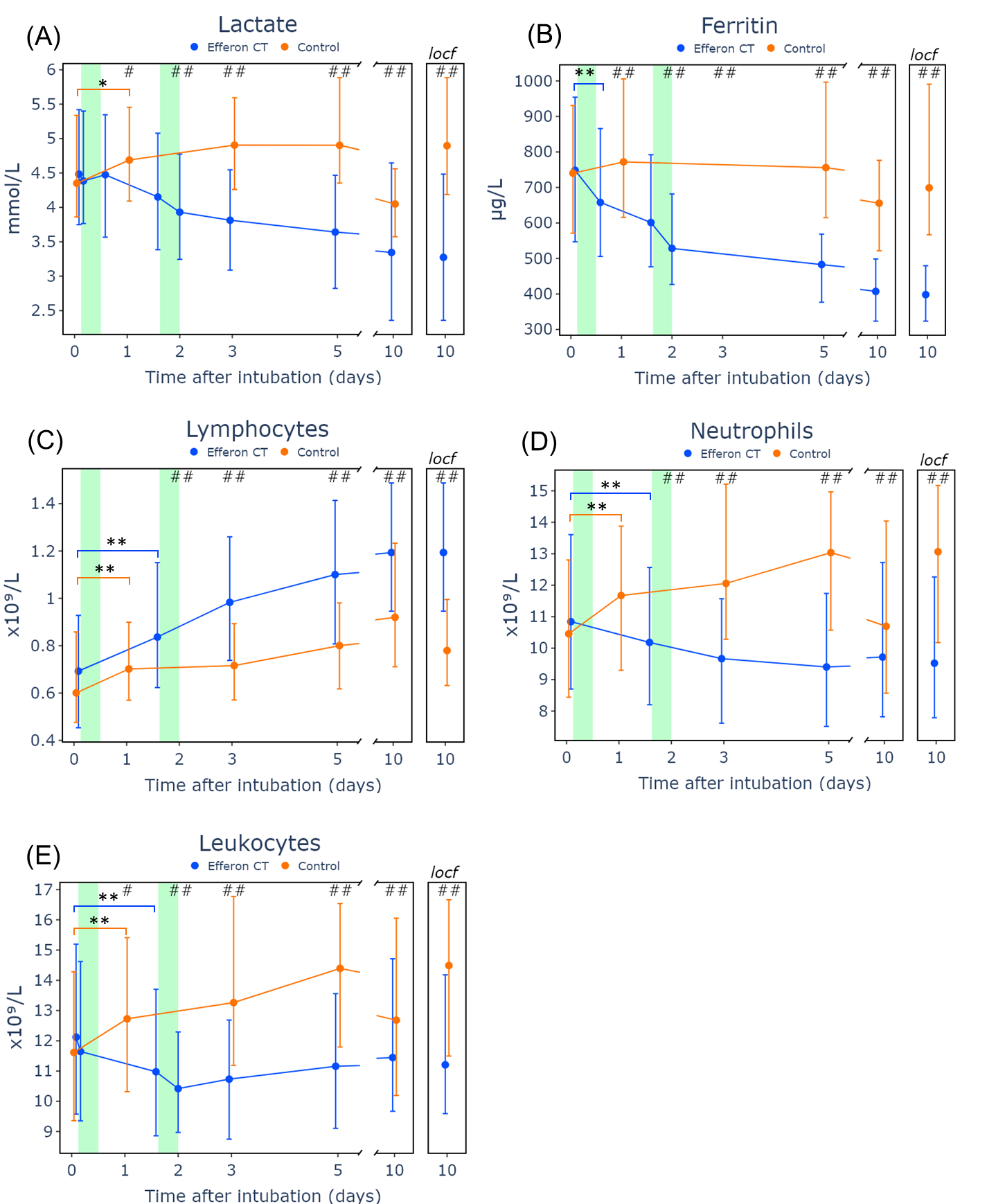


**Figure S2.** Dynamics of lactate (A), ferritin (B) and immune cells (C – E). Data are presented as Me (IQR). # *p* < 0.05, ## *p* < 0.001, between-group Mann-Whitney U test. * *p* < 0.05, ** *p* < 0.001, within-group Wilcoxon signed-rank test.

**Table S1.** Major comorbidities at the time of inclusion in the study.

| Comorbidity | Efferon | Control | *p* - value |
| --- | --- | --- | --- |
| Hypertensive heart disease | 43/65 (66%) | 44/65 (67%) | 1 |
| Obesity | 14/65 (21%) | 13/65 (20%) | 1 |
| Coronary heart disease | 16/65 (24%) | 13/65 (20%) | 0.674 |
| Postinfarction cardiosclerosis | 6/65 (9%) | 3/65 (4%) | 0.492 |
| Urolithiasis | 3/65 (4%) | 3/65 (4%) | 1 |
| Hepatitis C | 2/65 (3%) | 1/65 (1%) | 1 |
| Non-specific ulcerative colitis | 2/65 (3%) | 0/65 (0%) | 0.496 |
| Stomach ulcer | 1/65 (0%) | 1/65 (1%) | 1 |
| Duodenal ulcer | 0/65 (0%) | 1/65 (1%) | 1 |
| Gastritis | 1/65 (1%) | 0/65 (0%) | 1 |
| Heart pacemaker | 0/65 (0%) | 1/65 (1%) | 1 |
| Coxarthrosis | 1/65 (1%) | 0/65 (0%) | 1 |
| Acute cerebrovascular accident in anamnesis | 0/65 (0%) | 1/65 (1%) | 1 |
| Post-cholecystectomy syndrome | 1/65 (1%) | 0/65 (0%) | 1 |
| Without comorbidities | 3/65 (5%) | 3/65(5%) | 1 |

**Table S2.** Sputum microorganisms of patients at the time of inclusion in the study.

| Sputum culture | Efferon CT group, n = 65 | Control group, n = 65 | *p* - value |
| --- | --- | --- | --- |
| Positive result | 36/65 (55%) | 27/65 (41%) | 0.16 |
| *Acinetobacter spp* | 10/65 (15%) | 13/65 (20%) | 0.646 |
| *Enterobacter spp.* | 10/65 (15%) | 7/65 (4%) | 0.321 |
| *Klebsiella pneumoniae* | 3/65 (5%) | 0/65 (0%) | 0.244 |
| *Staphylococcus aureus* | 4/65 (6%) | 3/65 (5%) | 1 |
| Methicillin-resistant *Staphylococcus epidermidis* | 1/65 (2%) | 1/65 (2%) | 1 |
| *Escherichia coli* | 6/65 (9%) | 3/65 (5%) | 0.492 |
| *Pseudomonas aeruginosa* | 4/65 (6%) | 3/65 (5%) | 1 |

Note: Data are presented as n/N (%) where n is the number of patients with positive results, N is the total number of patients. *p*-value was calculated using Fisher's exact test.

**Table S3.** Presumable prevalence of SARS-CoV-2 variants in this study.

| variant/sub-variants of the SARS-CoV-2 | Efferon CT group | Control group |
| --- | --- | --- |
| Wuhan + Alpha  (B.1 + B.1.1* + B.1.1.7) | 50 – 60% | 48 – 57% |
| Delta +  (B.1.617.2 + AY*) | 15 – 25% | 16 – 26% |
| Omicron  (B.1.1.529 + BA* + BF* + BE*) | 22 – 28% | 23 - 29% |

^*^The estimation was conducted by analyzing epidemiological data from both regional sources^1,2^ and statistical data collected at the research center, while also taking into account the enrollment rate in this study.

^1^ Gushchin V.A., Pochtovyi A.A., Kustova D.D., Ogarkova D.A., Kleymenov D.A., Semenenko T.A., Logunov D.Yu., Zlobin V.I., GintsburgA.L. Characterisation of the COVID-19 epidemic process in Moscow and search for possible determinants of the trends of the observed changes. Journal of microbiology, epidemiology and immunobiology.2023;100(4):267–284. DOI: <https://doi.org/10.36233/0372-9311-375>.

^2^ Akimkin V.G., Semenenko T.A., Ugleva S.V., Dubodelov D.V., Kuzin S.N., Yacyshina S.B., Khafizov K.F., Petrov V.V., Cherkashina A.S., Gasanov G.A., Svanadze N.K. COVID-19 in Russia: Epidemiology and Molecular Genetic Monitoring. Annals of the Russian academy of medical sciences. - 2022. - Vol. 77. - N. 4. - P. 254-260. Doi: [10.15690/vramn2121](https://doi.org/10.15690/vramn2121)

**Definitions**

**MLV-free days** at 60-day period are defined as follows:

• MLV-free days = 0 if subject dies within 60 days of mechanical ventilation.

• MLV-free days = 60 − x if successfully liberated from ventilation x days after initiation.

• MLV-free days = 0 if the subject is mechanically ventilated for > 60 days.

ICU-free days and hospital-free days were defined in the same manner.

1.

Yehya, N.; Harhay, M.O.; Curley, M.A.Q.; Schoenfeld, D.A.; Reeder, R.W. Reappraisal of Ventilator-Free Days in Critical Care Research. *Am J Respir Crit Care Med* **2019**, *200*, 828–836, doi:[10.1164/rccm.201810-2050CP](https://doi.org/10.1164/rccm.201810-2050CP).

**Vasoactive-inotropic score (VIS):**VIS2020 =

10,000 × Vasopressin dose (U/kg/min) +

100 × Epinephrine dose (pg/kg/min) +

100 × Norepinephrine dose (pg/kg/min) +

50 × Levosimendan dose (pg/kg/min) +

25 × Olprinone dose (pg/kg/min) +

20 × Methylene blue dose (mg/kg/h) +

10 × Milrinone dose (pg/kg/min) +

10 × Phenylephrine dose (pg/kg/min) +

10 × Terlipressin dose (pg/min) +

0.25 × Angiotensin II dose (ng/kg/min) +

Dobutamine dose (pg/kg/min) +

Dopamine dose (pg/kg/min) +

Enoximone dose (pg/kg/min)

Reproduced from Belletti, A.; Lerose, C.C.; Zangrillo, A.; Landoni, G. Vasoactive-Inotropic Score: Evolution, Clinical Utility, and Pitfalls. *Journal of Cardiothoracic and Vascular Anesthesia* **2021**, *35*, 3067–3077, doi:[10.1053/j.jvca.2020.09.117](https://doi.org/10.1053/j.jvca.2020.09.117).
